# Understanding Surface Modifications Induced via Argon Plasma Treatment through Secondary Electron Hyperspectral Imaging

**DOI:** 10.1002/advs.202003762

**Published:** 2021-01-04

**Authors:** Nicholas Farr, Jeerawan Thanarak, Jan Schäfer, Antje Quade, Frederik Claeyssens, Nicola Green, Cornelia Rodenburg

**Affiliations:** ^1^ Department of Materials Science and Engineering Sir Robert Hadfield Building University of Sheffield Mappin Street Sheffield S1 3JD UK; ^2^ Insigneo Institute for In Silico Medicine The Pam Liversidge Building Sir Robert Hadfield Building University of Sheffield Mappin Street Sheffield S1 3JD UK; ^3^ Leibniz Institute for Plasma Science and Technology (INP e.V.) Felix‐Hausdorff‐Str. 2 Greifswald 17489 Germany

**Keywords:** argon plasma treatment, polymer characterization, polymeric biomaterials, secondary electron emission, secondary electron hyperspectral imaging

## Abstract

Understanding the effects that sterilization methods have on the surface of a biomaterial is a prerequisite for clinical deployment. Sterilization causes alterations in a material's surface chemistry and surface structures that can result in significant changes to its cellular response. Here we compare surfaces resulting from the application of the industry standard autoclave sterilisation to that of surfaces resulting from the use of low‐pressure Argon glow discharge within a novel gas permeable packaging method in order to explore a potential new biomaterial sterilisation method. Material surfaces are assessed by applying secondary electron hyperspectral imaging (SEHI). SEHI is a novel low‐voltage scanning electron microscopy based characterization technique that, in addition to capturing topographical images, also provides nanoscale resolution chemical maps by utilizing the energy distribution of emitted secondary electrons. Here, SEHI maps are exploited to assess the lateral distributions of diverse functional groups that are effected by the sterilization treatments. This information combined with a range of conventional surface analysis techniques and a cellular metabolic activity assay reveals persuasive reasons as to why low‐pressure argon glow discharge should be considered for further optimization as a potential terminal sterilization method for PGS‐M, a functionalized form of poly(glycerol sebacate) (PGS).

## Graphical abstract

A novel technique built upon secondary electron spectroscopy (SES) provides a detailed investigation into the effects argon plasma treatment has on a biomaterial's surface structures. This unique approach allows for key insights into a samples emission properties and in turn their chemical functional groups, which results in high resolution chemical imaging.

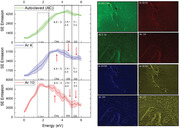

Biomaterials are rapidly expanding their contribution to today's medical progress, particularly in the role of a permanent or transient alternative for damaged or diseased tissues. It is essential that all biomaterials are sterilized prior to implantation within the patient. Additionally, the preservation of the mechanical properties and surface chemistry of a biomaterial poststerilization is essential for the correct function of the material within the body.^[^ ^1^ ^]^ However, commonly deployed sterilization techniques such as autoclaving (AC), irradiation, or chemical treatment have been shown to alter the surface and/or the mechanical structure of polymer‐based biomaterials.^[^ ^2^ ^]^ In comparison, plasma technology has demonstrated a promising potential for surface treatment of biomaterials.^[^ ^3^ ^]^ A recent review suggests that argon (Ar) plasma can be employed as a viable alternative sterilization procedure for biomaterials.^[^ ^4^ ^]^ However, several challenges have to be overcome in order to establish plasma sterilization as a standardized ISO method including the characterization of bioactive species of plasma,^[^ ^5^ ^]^ understanding of microbiological interactions,^[^ ^6^ ^]^ and achieving standardization of the plasma sterilization method. In particular, ISO standardization requires separate time consuming analyses of surface properties for each specified sterilization condition. Here, Ar plasma treatment is combined with the deployment of semi gas‐permeable packaging in order to form a model treatment of potential sterilization method for future use with polymer‐based biomaterials. Moreover, secondary electron hyperspectral imaging (SEHI) is applied to reveal any localized changes in key functional groups (CH, OH, and CO) on the surface of a biomaterial induced as a result of Ar treatment.

The foundation of SEHI is the collection of secondary electron (SE) emission spectra by means of the scanning electron microscope (SEM). The SE spectra for some hydrocarbon materials were found to be strongly influenced by excitation of intramolecular vibrations.^[^ ^7^ ^]^ Thus, SEHI can be successfully applied to the characterization of polymers,^[^ ^8^ ^]^ including novel polymeric biomaterials, and has already revealed the changes in molecular weight and CH vibrations that occur as a consequence of Ar plasma treatment.^[^ ^9^ ^]^ As SEHI is carried out in the SEM, local variation in chemical changes and sample topography can be assessed in one step.^[^ ^10^ ^]^ To do this, SEHI constructs a series of images of the sample area, where each image is formed from a selected SE energy band. While innovative synchronized structural and chemical characterization of materials by SEHI has many uses, this study focuses on revealing and mapping the spatial variation of functional groups that are affected by Ar plasma treatment through identifying specific energy bands for CH—, OH—, and CO— groups. In order to identify suitable energy bands for mapping such variations, a number of reference materials are used to evaluate the effects that Ar plasma treatment exhibits on local surface chemistry of sterilized biomaterials. This is important as a biomaterial's functional groups are closely coupled to its ability to promote cell growth through the local surface variations that result from the differing polymer side chains.^[^ ^11^, ^12^, ^13^ ^]^ Detrimental surface hydrophobic characteristics of some biomaterials developed from synthetic polymers are manifested as a lack of cell adhesion,^[^ ^14^ ^]^ resulting in the implanted biomaterial presenting poor tissue infiltration and integration outcomes. Thus a range of material characterization techniques in addition to SEHI are utilized here to further evaluate the effects of Ar plasma treatment of the surface topology of a polymer‐based biomaterial, in this instance PGS‐M.

PGS‐M is a functionalized form of poly(glycerol sebacate) (PGS)^[^ ^15^, ^16^ ^]^ The PGS‐M polymer, formed by rendering PGS (an elastomeric degradable and nontoxic tunable polymer^[^ ^15^ ^]^) photocurable through the process of methacryation, displays characteristics that are perceived as advantageous for biomaterial applications.^[^ ^17^, ^18^ ^]^ It has been observed that PGS‐M is likely to include small quantities of unreacted methacrylate side chains after polymerization by UV, removal of the unreacted methacrylate, and creating OH— groups on the surface has the potential to aid cellular growth and further the materials biocompatibility. Previous studies have indicated that plasma treatment can remove unreacted methacrylate.^[^ ^19^ ^]^ In this study, low‐pressure argon glow discharge has been applied for surface treatment of PGS‐M samples. Plasma treated samples exhibit different functional group densities than those of nontreated PGS‐M control samples. Changes include CO bonding and an increase of polar hydroxyl groups established through SEHI. These changes explain the varying ability of PGS‐M to support cellular adhesion after AC or Ar plasma treatment as demonstrated by contact angle measurements and cell metabolic activity assays.

The PGS‐M materials were enclosed in a gas semipermeable bag, which was exposed to low‐pressure Ar glow discharge. Use of the semipermeable bag ensures that any sterilization effect will not be compromised between the Ar plasma treatment and cell growth experiments. In order to assess the chemical changes taking place during this Ar plasma treatment, and compare this to the effects of AC sterilization, SE spectra were collected and presented in **Figure** **1**. This figure shows the SE spectra of AC PGS‐M, 10 min argon plasma treated PGS‐M (Ar 10), and 4 min argon plasma treated PGS‐M (Ar 4) (nontreated PGS‐M spectra can be found in the Supporting Information). For all treatments a peak is observable within the 1.4–2.3 eV range, however, differences arise in intensities of the peaks found within this range. Previous studies have isolated the energy range of 1.4–2.3 eV to the molecular order of carbon (C 1) polymers^[^ ^6^ ^]^ with a higher order yield intensity in this energy region. Ar 10 clearly exhibits a greater carbon peak (*M*_w_) compared to that of Ar 4 and AC. This can be understood as follows: argon plasma treatment causes a high levels of free radicals to create crosslinking sites post‐treatment, which directly affect the molecular order of the material. Argon plasma treated surfaces also exhibit a greater SE emission in the energy range that is associated with CH vibrations, with all samples expressing CH_2_ and CH_3_ peaks (Rationale in Figure S2 in the Supporting Information). Furthermore, the intensity is increased by prolonged Ar plasma exposure. The peak intensity in this energy region has been previously shown to have a direct relationship with the crosslinking density of PGS‐M.^[^ ^9^ ^]^ Therefore, the SE spectrum of Ar 10 suggests a highly crosslinked surface layer. Reactions within the surface structure by argon plasma induced radicals form crosslinks, which have the potential to enhance the crosslinking density between the surface molecular chains.^[^ ^20^, ^21^ ^]^

**Figure 1:**
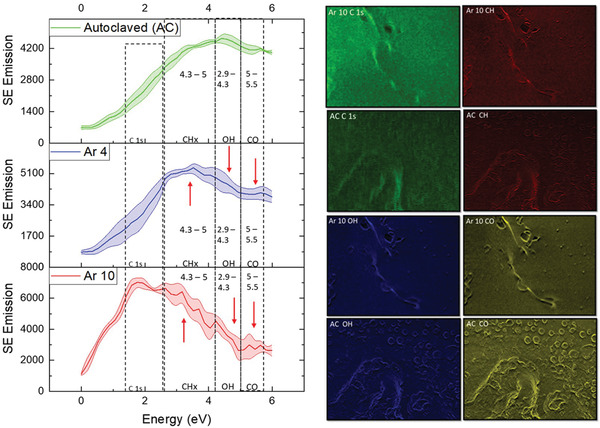
A) Secondary electron spectra for AC, Ar 4, and Ar 10 treated PGS‐M highlighting the regions identified as associated with functional group emissions. B) SEHI images generated from the component analysis of Ar 10 and AC. Mapping C 1s, CH, OH, and CO bonding.

Further, differences in the SE spectra for argon plasma treated and AC samples are observed within the region of 5–5.5 eV, related to C=O bonding (Figure S2, Supporting Information). The spectrum shows that post‐argon plasma treatment emissions in the 5–5.5 eV range are greatly diminished. Argon plasma treatment is understood to cleave away C—O—C bonds attached to the methacrylate within PGS‐M (Figure S6, Supporting Information). By cleaving away this bond, removal of methacrylate greatly decreases the amount of C=O bonds present within the polymer. It is worth noting that the decrease in C=O bonding is most noticeable in the Ar 10 rather than Ar 4 samples, which indicates that the cleavage of methacrylate units is time and area dependent. As more methacrylate is removed, surface crosslinking recombination increases, resulting in an increase in surface crosslinking of Ar 10. Once methacrylate units are removed, an OH containing hydroxyl group remains, signified by Ar 10 and Ar 4 showing emissions within 4.3–5 eV.

While the SE spectra provide an insight into the average chemical changes at the surface, which could also be obtained by other surface sensitive methods such as x‐ray photoelectron spectroscopy (XPS) (Figures S3–S5, Supporting Information), using the above specific energy ranges to form images in the SEM is only possible with SEHI. In order to obtain images from the corresponding spectral components, a non‐negative matrix factorization (nnmf) was used to isolate various components between 0 and 6 eV (Figure S7, Supporting Information). Figure 1B displays images generated of these components from the Ar 10 and AC surfaces that can be matched to the peak allocation described in the Supporting Information (SEHI images and nnmf of nontreated PGS‐M are displayed in Figure S13 in the Supporting Information). When viewing SEHI images, the brighter the primary color, the greater the SE emissions associated with that component. Consequently, the brighter the emission color, the greater the functional group prevalence,^[^ ^9^ ^]^ where all analyzed materials are used to determine the upper and lowest emission values. It is expected that at the length scale of micro/nanometers, structures will include emission from multiple functional groups. To establish the distribution of functional groups, one should consider the brightness of specific emissions. Figure 1B shows the distribution of C 1, C—H, O—H, and C=O bonding within Ar 10 and AC samples.

It is notable that the topography of Ar 10 plasma treated samples differs greatly from AC samples, an observation consistent with previous reports.^[^ ^4^ ^]^ SEHI images show micrometer‐sized spherical features are clearly present within AC, with strong SE emission signatures of C=O bonding. The C=O bonding related emissions of these structures indicate that they contain excess methacrylate groups. C=O bonding across the matrix of Ar 10 on the whole is diminished seemingly as a result of the cleaving of methacrylate post‐Ar plasma treatment. SEHI images displayed in Figure 1B do show spherical nanofeatures within Ar 10, which exhibit emission of C=O bonding. It is proposed that remnants of methacrylate, which have not yet been cleaved away from the surface of PGS‐M by Ar plasma treatment, are still traceable. Additionally, the micrometer spherical features within AC samples emitted a far weaker CH bonding signature than the Ar plasma treated samples, indicating that less crosslinking occurs around these larger regions. **Figure** **2** displays SEHI images that further confirm the breakdown of the micrometer spherical features in AC PGS‐M with high SE emission in relation to CO bonding into the nanodot structures present in the Ar 10 surface.

**Figure 2:**
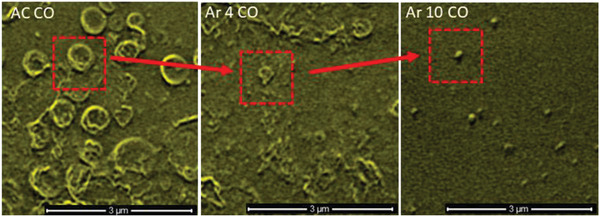
SEHI images generated from the component analysis of AC, Ar 4, and Ar 10 mapping CO bonding. Red insets highlight the reduction of micrometer–nanoscale features high in CO bonding.

From SEHI images alone it is obvious from the reduction of area with strong SE emission in the CO— bonding related energy range that excess methacrylate has been removed from the sample as a consequence of the Ar plasma treatment. Furthermore, the increase in OH groups and CH related crosslinking has clearly become more abundant across the surface matrix post‐Ar plasma treatment. As previously stated, the removal of unreacted methacrylate together with the introduction of surface OH groups is recognized to aid cell growth through the enhanced cellular response expected in materials that exhibit greater crosslinking, and it is expected that cells would adhere and proliferate better on Ar plasma treated surfaces.

To test the expectations based on SEHI functional group mapping and for the purpose of using argon plasma as a terminal sterilant, it is important to understand how the SEHI results link to those obtained through commonly practiced characterization methodologies. This is achieved by investigating how the argon plasma treatment has affected the mechanical properties of PGS‐M. **Figure** **3**A shows the results of nanoindentation. As forecasted by the SEHI analysis above, Ar 10 displayed an increase in hardness compared to Ar 4 or AC samples. The trend in the nanoindentation data substantiates the SEHI map of CH vibrations and opens up the potential of SEHI as a nondestructive alternative to time consuming nanoindentation measurements. Furthermore, SEHI has the advantage of allowing for crosslinking information to be obtained at a scale smaller than that available with nanoindentation, using CH‐bonding maps.

**Figure 3:**
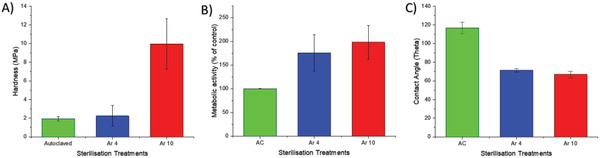
A) The hardness (MPa) obtained from nanoindentation for varying degrees of AC, Ar 4, and Ar 10 treated PGS‐M (mean ± STD error bars). B) The cell metabolic rate increase (Resazurin assay) of cells cultured on argon treated PGS‐M with relative increase given compared to that of AC scaffolds C) The contact angle (theta) obtained for varying degrees of AC, Ar 4, and Ar 10 treated PGS‐M (mean ± STD error bars).

While CH‐bonding maps offer a potential substitute for nanomechanical testing, we speculate that OH‐bonding maps could substitute for contact angle measurements since cleavage of excess methacrylate units from PGS‐M allows the formation of hydroxyl groups, leading to an increased hydrophilicity. SEHI bonding maps allow for spatially detailed information unlike that from a contact angle, which just gives bulk samples data. XPS data (Figure S3, Supporting Information) and SE spectra (Figure 1A) both show the average content of OH— groups increase as a result of Ar treatment. What SEHI offers is a greater spatial understanding of how the micrometer–nanoscale structures emit high traces of OH— groups in the AC samples. SEHI images in Figure S9 in the Supporting Information show that the Ar plasma treated PGS‐M displayed a greater signature of OH‐groups around nanoscale structures, which are polar and therefore increase hydrophilicity.^[^ ^22^ ^]^ Indeed, contact angle results (Figure 3C) showed that argon plasma treatment increased the hydrophilicity, which is consistent with previous studies that demonstrated that plasma has the capacity to change the surface chemistry, generating increased hydrophilicity and resulting in enhanced cell adhesion.^[^ ^23^ ^]^ However, it is noted that there can be contributions from topography changes, which can effect contact angle measurements as surface alterations are visible. Previous research has also shown that an increase in OH containing functionalities is proportional to improved cellular growth.^[^ ^24^ ^]^ Based on this and SEHI results, one expects improved cellular growth even though the sample materials were not directly exposed to the plasma but were enclosed within a gas‐permeable bag ensuring that the surfaces remain sterile after plasma treatment until future use.

Here, we assess the sample materials potential to support cellular growth using a cellular metabolic activity assay (Resazurin assay). Figure 3B displays metabolic activity of cells growing on the argon plasma treated PGS‐M samples and also the AC PGS‐M samples. The results show greater cellular metabolic activity on the argon plasma treated sample surfaces, indicative of an increase in cell adherence and proliferation above that of the AC samples. Although a large disparity in cellular metabolic activity was observed between the argon plasma treated samples, it is considered that this was due to variation in plasma conditions within the actual plasma chamber at different sample positions due to the Ar inlet being located at the rear of the plasma chamber. These disparities emphasize the importance of minimizing variations in the plasma conditions, a more consistent plasma environment may be achievable using more advanced plasma sources.

The observed data confirms that argon plasma treatment shows a positive relationship to cellular growth. However, SEHI data and nanoindentation also identified that Ar plasma treatment significantly increased the stiffness of PGS‐M samples due to crosslinking. SEHI maps of CO bonding suggest that surface structures vary locally and can be changed from ≈500 nm size (4 min Ar) to ≈50 nm size (10 min Ar) depending on Ar treatment time. Therefore, future work is recommended to optimize the Ar plasma intensity and duration in order to produce a sterilized biomaterial while controlling the local variation of mechanical properties to surface patterns that promote cellular growth. Further to this work, a large scale sterilization study is required to determine the efficacy of the model argon plasma treatments with regards to sterilization at various conditions with a view to providing evidence of its capacity to achieve the recommended sterility assurance levels (SALs) to be termed a sterilization method under ISO requirements^[^ ^25^, ^26^ ^]^ SEHI offers an efficient way to achieve this due to its ability to map functional groups with the required image resolution together with an ability to map at multilength scales. This ability is vital to attaining a comprehensive understanding of cell behavior on Ar plasma treated surfaces. This future work, in combination with the use of gas semipermeable bags as demonstrated in this study, would pave the way for the wider deployment of argon plasma as a terminal biomaterial sterilization process.

## Conflict of Interest

The authors declare no conflict of interest.

## Supporting information

Supporting Information

## References

[1] I. Chiulan , A. N. Frone , C. Brandabur , D. M. Panaitescu , Bioengineering 2017, 24, 5. 10.3390/bioengineering5010002 PMC5874868 29295559

[2] M. Ahmed , G. Punshon , A. Darbyshire , A. M. Seifalian , J. Biomed. Mater. Res., Part B 2013, 101, 1182. 10.1002/jbm.b.32928 PMC4228764 24039066

[3] K. Schröder , A. Meyer‐Plath , D. Keller , A. Ohl , Plasmas Polym. 2002, 7, 103.

[4] M. Griffin , N. Naderi , D. M. Kalaskar , E. Malins , R. Becer , C. A. Thornton , I. S. Whitaker , A. Mosahebi , P. E. M. Butler , A. M. Seifalian , Int. J. Biomater. 2018, 2018, 6565783. 30405715 10.1155/2018/6565783 PMC6192142

[5] X. Lu , G. V. Naidis , M. Laroussi , S. Reuter , D. B. Graves , K. Ostrikov , Phys. Rep. 2016, 630, 1.

[6] P. Bourke , D. Ziuzina , L. Han , P. J. Cullen , B. F. Gilmore , J. Appl. Microbiol. 2017, 123, 308. 28245092 10.1111/jam.13429

[7] N. A. Stehling , R. Masters , Y. Zhou , R. O'Connell , C. Holland , H. Zhang , C. Rodenburg , MRS Commun. 2018, 8, 226.

[8] R. C. Masters , N. Stehling , K. Abrams , V. Kumar , A. Schäfer , D. Lidzey , C. Rodenburg , Adv. Sci. 2019, 6, 1801752. 10.1002/advs.201801752 PMC6402282 30886802

[9] N. Farr , S. Pashneh‐Tala , N. Stehling , F. Claeyssens , N. Green , C. Rodenburg , Macromol. Rapid Commun. 2019, 41, 3. 10.1002/marc.201900484 31859420

[10] K. J. Abrams , M. Dapor , N. Stehling , M. Azzolini , S. J. Kyle , J. S. Schäfer , A. Quade , F. Mika , S. Kratky , Z. Pokorna , I. Konvalina , D. Mehta , K. Black , C. Rodenburg , Adv. Sci. 2019, 6, 1900719. 10.1002/advs.201900719 PMC6774015 31592411

[11] J. N. Barbosa , P. Madureira , M. A. Barbosa , A. P. Aguas , Biomaterials 2005, 26, 3021. 15603797 10.1016/j.biomaterials.2004.09.002

[12] S. N. Rodrigues , I. C. Gonçalves , M. C. Martins , M. A. Barbosa , B. D. Ratner , Biomaterials 2006, 27, 5357. 16842847 10.1016/j.biomaterials.2006.06.010

[13] Y. Arima , H. Iwata , Biomaterials 2007, 28, 3074. 17428532 10.1016/j.biomaterials.2007.03.013

[14] J. N. Lee , X. Jiang , D. Ryan , G. M. Whitesides , Langmuir 2004, 20, 11684. 15595798 10.1021/la048562+

[15] Y. Wang , G. A. Ameer , B. J. Sheppard , R. Langer , Nat. Biotechnol. 2002, 20, 602. 12042865 10.1038/nbt0602-602

[16] S. Pashneh‐Tala , R. Owen , H. Bahmaee , S. Rekštyte , M. Malinauskas , F. Claeyssens , Front. Phys. 2018, 6, 41.

[17] M. Kharaziha , M. Nikkhah , S. R. Shin , N. Annabi , N. Masoumi , A. K. Gaharwar , G. Camci‐Unal , A. Khademhosseini , Biomaterials 2013, 34, 6355. 23747008 10.1016/j.biomaterials.2013.04.045 PMC3685203

[18] K.‐W. Lee , D. B. Stolz , Y. Wang , Proc. Natl. Acad. Sci. USA 2011, 108, 2705.21282618

[19] C. Liu , N.‐Y. Cui , S. Osbeck , H. Liang , Appl. Surf. Sci. 2012, 259, 840.

[20] F. Ayhan , H. Ayhan , E. Piskin , J. Bioact. Compat. Polym. 1998, 13, 65.

[21] S. Makoto , I. Taku , K. Masaru , N. Nobuyuki , T. Yasuhiko , Y. Hiroshi , J. Micro/Nanolithogr. 2013, 12, 41309.

[22] B. G. Keselowsky , D. M. Collard , A. J. García , Biomaterials 2004, 25, 5947. 15183609 10.1016/j.biomaterials.2004.01.062

[23] Y. Tamada , Y. Ikada , Polymer 1993, 34, 2208.

[24] S. I. Ertel , B. D. Ratner , T. A. Horbett , J. Biomed. Mater. Res. 1990, 24, 1637. 2148939 10.1002/jbm.820241207

[25] ANSI/AAMI ST67:2003 , Sterilization of health care products ‐ Requirements for products labeled “STERILE”.

[26] ANSI/AAMI/ISO 11137:2006 , Sterilization of health care products”.

