# Supplementary material for: Understanding Surface Modifications Induced via Argon Plasma Treatment through Secondary Electron Hyperspectral Imaging
Source: Adv Sci (Weinh). 2021 Jan 4;8(4):2003762. doi: 10.1002/advs.202003762 (PMC7887591; doi:10.1002/advs.202003762)
Supplement: Supplementary file 1 — Supporting Information [file ADVS-8-2003762-s001.pdf]

© 2020 Wiley-VCH GmbH

ADVANCED SCIENCE
Open Access

Supporting Information

for *Adv. Sci.,* DOI: 10.1002/advs.202003762

Understanding surface modifications induced via Argon Plasma treatment through Secondary Electron Hyperspectral Imaging

Nicholas Farr, Jeerawan Thanarak, Jan Schäfer, Antje Quade, Frederik Claeyssens, Nicola Green, and Cornelia Rodenburg*

Supplementary Information

Understanding surface modifications induced via Argon Plasma treatment through Secondary Electron Hyperspectral Imaging

Nicholas T.H Farr, *[1,3] Jeerawan Thanarak, [1,3] Jan S. Schäfer, [2] Antje Quade, [2] Frederik Claeyssens, [1] Nicola H. Green, [1,3] and Cornelia Rodenburg[1]

1 Department of Materials Science and Engineering, Sir Robert Hadfield Building, Mappin Street, University of Sheffield, UK.

2. Leibniz Institute for Plasma Science and Technology. (INP Greifswald e.V.) Felix-Hausdorff-Str. 2, 17489 Greifswald, Germany.

3. Insigneo Institute for *in silico* Medicine, The Pam Liversidge Building, Sir Robert Hadfield Building, Mappin Street, Sheffield, UK

Materials and Methods

For the following methods, all chemical reagents were obtained from Sigma Aldrich, UK, unless otherwise stated.

Synthesis of polyglycerol (sebacate)-methacrylate (PGS-M)

The low molecular weight PGS-M polymer was fabricated following the protocol from Pashneh-Tala et al [1]. In brief, the PGS prepolymer was synthesised by mixing 1:1 (mol/mol) glycerol and sebacic acid, using a hot plate at 120°C, 300 rpm for 48 hours. Nitrogen gas was applied in the first 24 hours, then a vacuum was applied to the system for another 24 hours to remove the water from condensation. To methacrylate the PGS prepolymer, 1:4 (w/v) dichloromethane (DCM) was used to dissolve the prepolymer. Subsequently, the system was changed to 0°C in dark condition at 300 rpm. 1:1 (mol/mol of PGS hydroxyl groups) of Triethylamine (TEA) and 1 mg/g PGS hydroxyl group of 4-Methoxyphenol (MeHQ) were added into the system. Methacrylate anhydride (MAA) was used to control the percentage of methacrylation, in this case, 0.5 mol of MAA was added per mol PGS hydroxyl groups. After 24 hours of methacrylation, 30mM hydrochloric acid was used to wash the PGS-M polymer. The water from reaction was then removed by using $CaCl_2$. Lastly, DCM was removed by rotary evaporation.

To synthesis PGS-M scaffolds, residual DCM was taken out from PGS-M polymer using vacuum. 70% PGS-M in DCM was blended with 1:1 (w/w) toluene, 10% Hypermer^{TM} B246 and 25% diphenyl(2,4,6-trimethylbenzoyl) phosphine oxide/ 2-hydroxy 2-methylpropiophenone and blended (photoinitiator) at 350 rpm. After 5 minutes of blending, 4 ml dH_2O was added dropwise to the emulsion. The emulsion was then photocured for 5 minutes each side and washed with methanol for 4 days and dH_2O for 4 days.

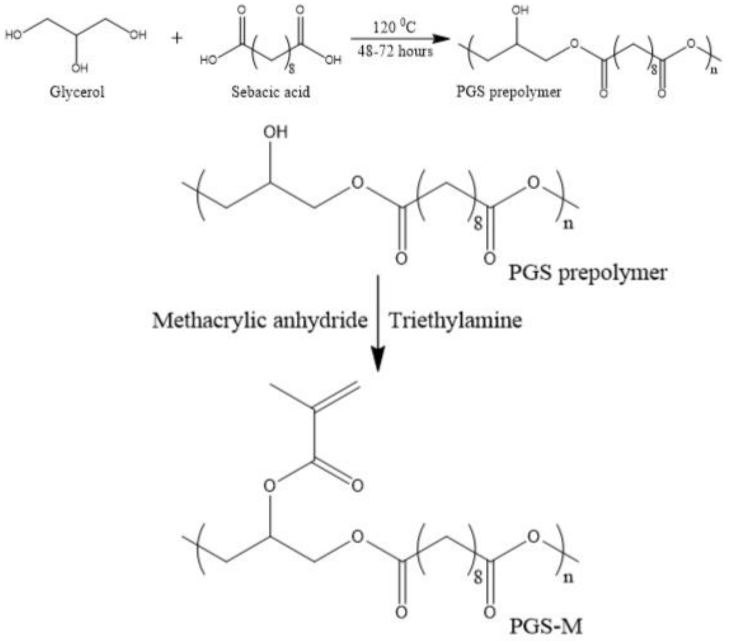

Figure S1 - PGS-M chemical synthesis from monomers, to PGS pre-polymer and finally produced of PGS-M.

Low Temperature Plasma Sterilisation Treatment

PGS-M samples were prepared by exposing them to low-pressure argon glow discharge in a Diener Electronic Zepto plasma cleaner at 40 kHz, 100 W, and 0.3 mbar air for 4 minutes and 10 minutes in Tyrex gas semi-permeable packaging. 10 minutes of exposure was chosen as a maximum limit to exclude any effects of sample heating which have been shown to lead to additional surface modifications [2].

Autoclave sterilisation

To sterilise PGS-M samples by steam sterilisation an autoclave method was performed. This required the samples to be immersed in dH2O within a hot-steam environment at approximately 121°C for 20 minutes. The autoclaved PGS-M samples were then stored in the sterile dH_2O.

PGS-M Characterisation

1. SEM and Image Data Processing

Imaging

Observation of the surface morphology of the PGS-M samples was performed using a Scanning Electron Microscope (FEI Nova Nano 450 SEM). The PGS-M samples were not subject to deposition of conductive coating, in contrast to usual polymers SEM analysis practice. To avoid surface charging and consequent damage to the sample a low accelerating voltage (1 KV) with typical vacuum pressure of 10^{-5} mbar at a working distance of 3mm was applied. An Everhart-Thornley Detector (ETD) for low magnification images and a Through Lens Detector (TLD) for high magnification images were used for the collection of SE images.

SEHI Acquisition and Image Processing

The FEI Nova Nano 450 SEM is provided with a through lens detector which includes a voltage controlled deflector electrode. The deflector electrode channels the signal into the SE detector. The deflector electrode is set to a predetermined number of deflector voltages and an image is generated for each deflector voltage. Spectra and hyperspectral images are acquired through post-processing of such image series. A short dwell time of 100 ns and the inclusion of 16 frame integrations is applied to reduce beam exposure and sample damage. Stage bias has been performed to allow energy calibration of this process through experiments [3, 4]. Fiji software was utilised to perform image processing [5]. Histogram and spectral off-set normalisation [6] has been applied retrospectively to optimise all images for brightness and contrast, and to limit the effects of differing sample work functions. Once S curves have then been obtained they are differentiated to produce the SE curves which are reported in the main manuscript. Component analysis was then preformed to image stacks utilising non negative matrix factorization (nnmf) to isolate components of interest.

Raman Spectroscopy

Raman spectroscopy (Renishaw inVia micro-Raman) was employed to analyse the chemical structure of the PGS-M placed on borosilicate glass. Using a 50x objective the laser power was 20 mW with a 1 µm spot size. A Peltier-cooled multichannel CCD detector was used for data recording with a 2,400 lines/mm diffraction grating at a slit opening of 65 µm and a spectral resolution of in the order of 1 cm^{-1}.

Nanoindentation

Nanoindentation measurement was performed on wax embedded PGS-M disks. The PGS-M was embedded at 42°C and sectioned smooth. A Bruker's Hysitron TI Premier nanoindenter, attached to a Berkovich tip was used for nanoindentation of PGS-M. A matrix of 12 indentations was applied, spaced 60 µm apart. The polymer sample was loaded for 5 s, held for

80 s, and unloaded for 3 s. The hold period was added to allow any effects from creep in the polymer to be minimised. A peak force of 200 µN was applied with a lift height of 20 nm. The Oliver Pharr method was utilised to analyse each of the unloading segments of the polymer indentation. This provided a reduced modulus (E_r) value as well as sample hardness (H).

Water contact angle

Before measuring water contact angle, PGS-M samples were air dried inside a class II biological cabinet. The wettability of a surface of the samples was measured by dropping 5 µl of water onto the dried surface. A blunt cannule was pointed vertically to the model and placed above the surface of the samples for approximately 1-2 mm before injecting the water droplet. The needle and the water droplet were monitored by the build-in camera. The horizontal line was specified at the upper surface of the sample. Once the water droplet was injected, the angle data and the image of the droplet were obtained within 30 seconds.

Cell Culture

Human dermal fibroblasts were isolated from donor skin obtained with informed consent and ethical approval (15/YH/0177). Fibroblasts were cultured in Dulbecco's Modified Eagle Medium (DMEM) with 10% (v/v) Fetal Calf Serum (FCS), $2x10^{-3}$ M glutamine, 10% (v/v) Penicillin-Streptomycin and 0.625 µg/ml amphotericin B. $1x10^{5}$. Cells were then seeded in a 12 well-plate and allowed to attach on the samples overnight before moving to a fresh 12 well-plate. The cells were then grown for one week at 37°C, 5% CO_2 in an incubator. A control was also prepared by seeding the cells onto tissue culture plastic surface. After one week of experiment, the samples were stained with resazurin assay to quantify the cell metabolic rate.

Metabolic activity assay

The reduction of resazurin to its fluorescent product resorufin was used to measure the metabolic activity of the cells on the scaffolds. Resazurin sodium salt solution (10 mg ml^{-1} in PBS) was diluted 1:50 ratio in fresh culture media. The cell seeded samples were then immersed in 2 ml of this solution for 4 hours in an incubator. The plate was placed on a rocker throughout the incubation time. Aliquots of the solution were then removed and fluorescent absorbance was measured using the Biotek FLx 800 fluorescence plate reader (λex 530 nm, λem 590 nm).

Statistical Analysis

SE spectra for all materials were processed through an in-house MATLAB script to organise and process the spectrum image stack in to the spectra given in Figure 1 and SI 2. Each materials SE spectra was formed by calculating a mean and

standard deviation of n=4 independent SE spectra measurements. An in-house Non-Negative Matrix Factorization (NNMF) method was applied to determine the spectrum distribution of the SE components within each material. NNMF retains the spatial information of extracted components that corresponds to the structural differences in the components. NNMF achieves this by allowing only positive correlated combinations which enables an intuitive representation of the data. NNMF spectra output is then presented graphically with no further statistical analysis.

Hardness measurements presented are set from the raw MPa hardness values provided by the Bruker's Hysitron TI Premier nanoindenter. To present these results in a graph, as shown in Figure 3, a mean and standard deviation was calculated from the n=12 raw indent measurements (captured for each material). For the cell metabolic assay, fluorescent absorbance was measured using the Biotek FLx 800 fluorescence plate reader. A mean and standard deviation of these values was then calculated and presented in figure 3. Contact angle (Theta) raw values was obtained and captured by the built-in camera. From the raw values captured a mean and standard division was calculated and presented in figure 3 for the three materials (n=3).

Additional Results

Analysis of Reference Samples

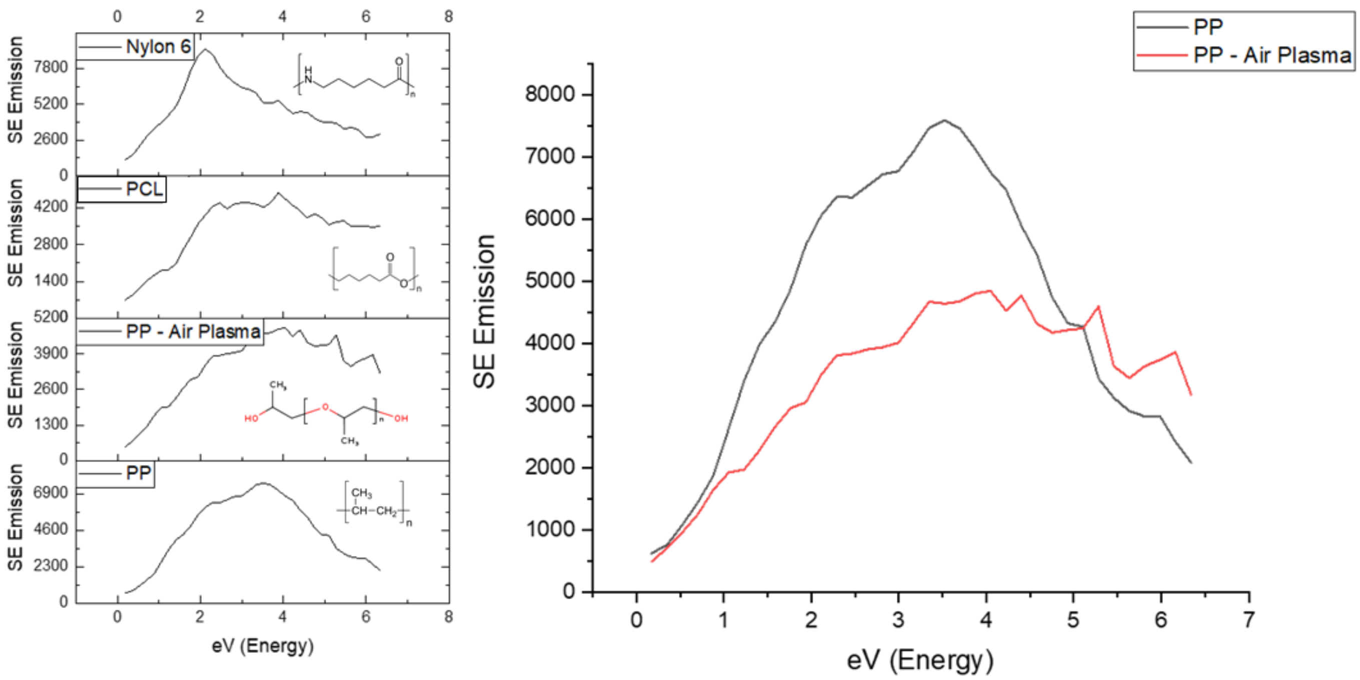

Figure S 2 - Secondary electron spectra for Nylon 6, Polycaprolactone, Polypropylene and Air Plasma treated Polypropylene.

Figure S2 displays the SE spectrums of Polypropylene (PP), Air Plasma treated PP, Nylon 6 and Polycaprolactone (PCL). Previous studies have isolated the energy range of 1.4 – 2.3 eV to the molecular order of the materials [7], for polymers, isolating the molecular weight of the material, thought to be associated with C-C backbone bonding [8]. For all reference materials (PP, PCL and Nylon 6) a peak is observable within the 1.4 – 2.3 eV range. Preceding studies have also characterised multiple peaks in this energy range of 2.9 - 4.3 eV as emissions attributed to CH vibrations [9]. In this instance two peaks occur within the data set. Firstly, a peak around 2.3 eV which is visible in all reference sample spectra, and secondly a peak around 3.2 eV which is expressed in all but Nylon 6. It is therefore considered that due the chemical structure of these materials the 2.3 eV peak is related to CH_2 bonding whereas the 3.2 eV peak is related to CH_3 bonding. This hypothesis explains why the 3.2 eV peak is absent in Nylon as this material does not possess the CH3 functional group.

Previously captured SE spectra of HOPG have shown that emission peaks around 4.5 – 5 eV are observed within aged HOPG [9]. It is now proposed that this is at least partially a consequence of oxidation on the material surface resulting in –OH hydroxyl groups forming. This process of oxidation to change the surface structure of a PP material was used in this study. The PP was air plasma treated to allow hydroxyl groups to form on the surface of the sample. During the air plasma

oxidation process polar groups such as carboxylic acids, ketones and ester groups will also develop at the polymer surface [10]. Figure S2 displays the SE spectra of air plasma treated PP and non-treated PP. It is notable that the molecular weight peak of air plasma treated PP is greatly reduced. This is attributed to surface melting occurring due to the slight surface heating associated with the action of the chemical etching breaking crosslinks, this may possibly also explain the reduction in CH vibrations observed. The air plasma treatment of PP is shown to have caused an increase in SE emissions within two peaks around 5 eV and 6 eV when compared to non-treated PP. The previously described 5 eV peaks in aged HOPG leads to an assumption that observations in this energy range are associated with an increase in OH groups on the surface of PP post aging oxidation. An additional peak of interest occurs around 6 eV, this peak is proposed to be the result of CO bonding ensuing from carboxylic acid groups that were formed post treatment.

To provide supporting evidence for this proposal, Nylon 6 and PCL-M were used as reference materials. Nylon 6 contains an amide CO, but does not possess a hydroxyl group. This is confirmed in its observed SE spectrum and further contributes to the proposal by showing emission peaks around 5 – 6 eV. However, there are minimal emission peaks present around 4.5 - 5 eV. PCL-M has emission peaks present at both 4.3-5 eV that are associated with OH and also at 5 – 6 eV which are considered to be associated with C=O (see figure 1D). This result is expected as PCL-M contains both of these functional groups.

X-ray photoelectron spectrometer (XPS) measurements of the samples were taken and applied to corroborate the SE spectra results. XPS is an effective technique for the identification of chemical composition and oxidation state. Subtle changes in peak positions and shape can yield information on changes in surface chemistry. Figure S3 shows all samples contained carbon, oxygen and nitrogen elements. The results indicated that all the samples surfaces showed evidence of impurities such as: (N), Si, Na, Ca, Cl and S. The samples also followed the same trends identified by SEHI in relation to O composition within their structures. Post plasma treatment the PP-surface showed a higher concentration of O in comparison to the original PP non-treated surface. The PP O/C ratio on treated sample surfaces was observed to increase after application of plasma treatment in air, a result that further points to plasma treatment establishing oxygen rich functional groups.

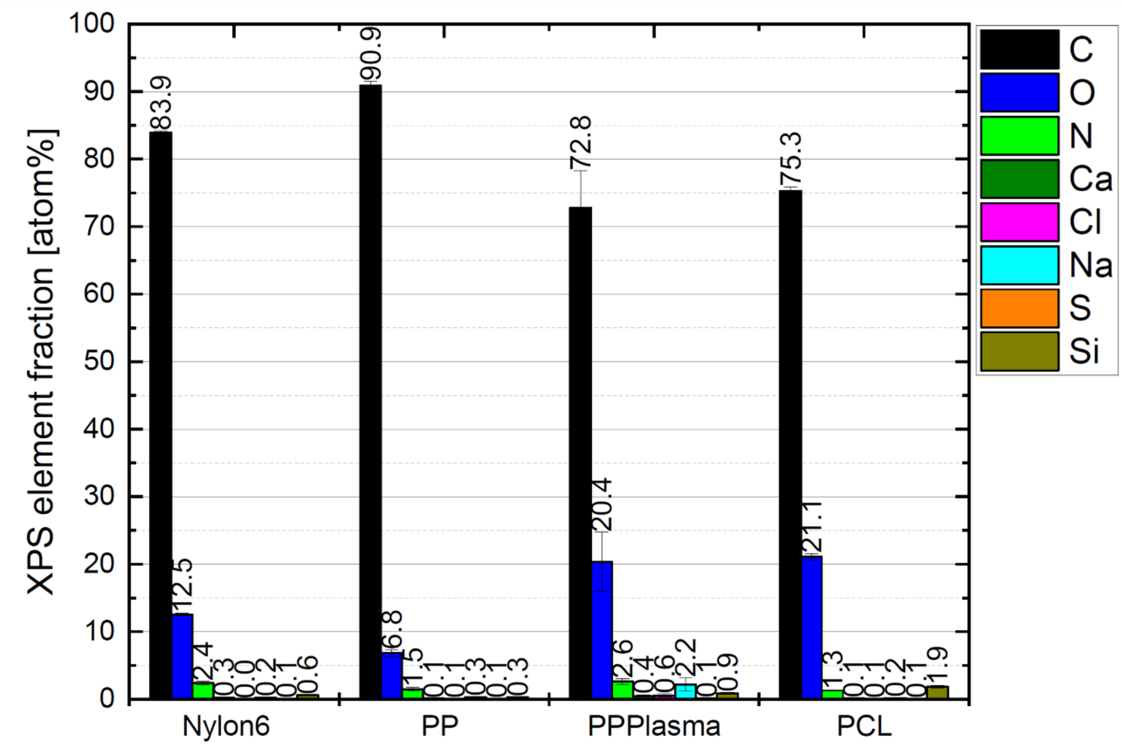

Figure S 3 - X ray photoelectron spectrometer (XPS) measurements of Nylon 6, PCL-M , Polypropylene and Air Plasma treated Polypropylene.

Survey scans of the non-treated and plasma treated polypropylene are shown in Figure S4. This figure displays that contaminates, such as: Na, N, Cl and Si were detectable, particularly after plasma treatment. Small peaks are noticeable within the accompanying SE spectra but further analysis is required before these can be reliably detected and characterised. The source of this contamination is expected to be the consequence of the multi sample use of the plasma chamber. Thus for sterilisation applications a dedicated plasma treatment chamber should be used.

Figure S5 depicts that plasma treated PP exhibiting a shape change of its XPS peak compared to that of untreated PP. The observable shoulders on the high bonding energy side of treated PP suggests that more O containing (and COOH/COOR @ 289.2 eV) functional groups were introduced by the plasma treatment of PP.

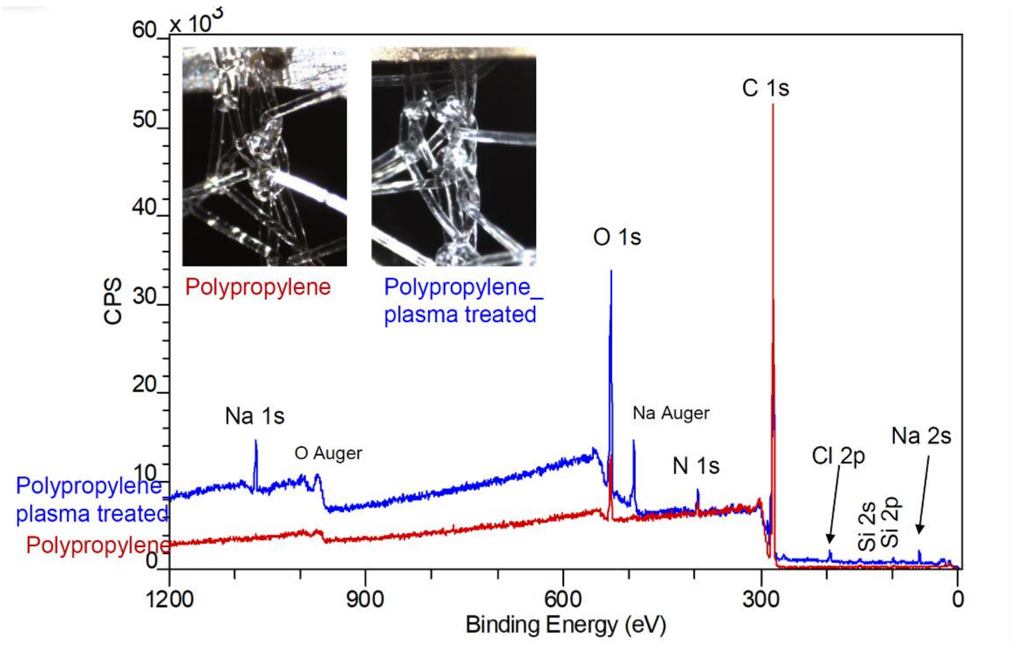

Figure S 4 - X ray photoelectron spectrometer (XPS) Survey scans of the non-treated and plasma treated polypropylene

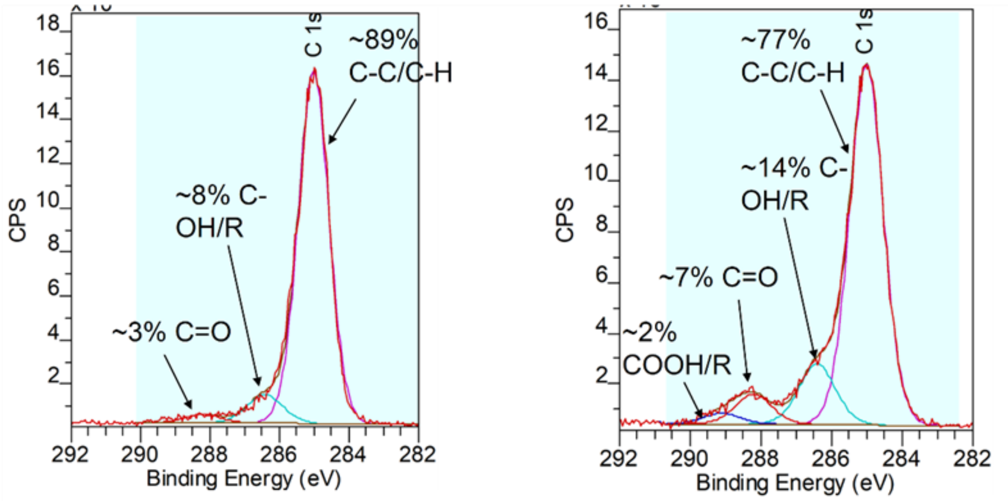

Figure S 5 - X ray photoelectron spectrometer (XPS) peak fits of the non-treated and plasma treated polypropylene

Further analysis of Argon Treated PGS-M

To support the premise that Argon plasma cleaves methacrylate from the surface of treated PGS-M, Raman spectra were captured from the samples (S6). Figure S6a shows the Raman fingerprint region in the range of 500 – 2000 cm^{-1}. A notable difference between Argon plasma treated and AC samples is observed within a peak at 1045 cm^{-1} , which is associated with C-O-C bonding in PLA [11]. SE analysis correlates with the observed CH vibrations (2750 – 3200 cm^{-1}) peak present in the Raman spectra as predicted by these results. The changes in Raman intensity at 2950 cm^{-1} (CH vibrations) closely correlates with the SE intensity changes within the 2.9 – 4.3 eV range. Both peaks are similarly affected by CH bonding and consequently the cross-linking process of PGS-M. The consistency of both SE and Raman spectra provides a robust argument that both techniques can detect cross-linking in PGS-M through CH bonding changes. However, SE displays the additional benefits of a multiscale imaging capability and thus the ability to capture spatial variations.

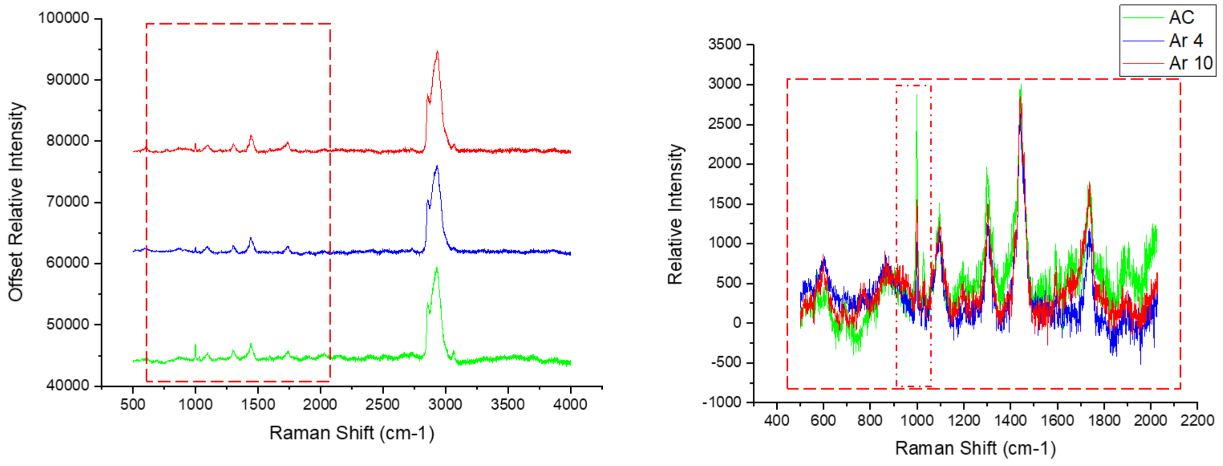

Figure S 6 - A) Off set full Raman spectrums for AC, AR 4 and Ar 10 treated PGS-M. B) Raman fingerprint region spectrums for AC, AR 4 and Ar 10 treated PGS-M.

From the combined results of Raman and SE analysis there is a strong argument that unreacted methacrylate has been removed by Argon plasma treatment. A slight reduction of C=O was identified by Raman (700 cm^{-1}). However, as the escape depth of Raman is greater it is likely Raman is giving reference to the aggregate sample whereas SEHI is closely focused at the surface of PGS-M with an escape depth of 10 nm.

In order to obtain images from the corresponding spectral components a non-negative matrix factorisation (nnmf) was used to isolate various components between 0 – 6 eV. Figure 1B displays images generated these components from the Ar 10 and AC surfaces that can be matched to the peak allocation described above. Of specific interest, one component

consisted of a peak ~3 eV highlighted that was previously identified as CH vibrations. Additionally, a component at 4.5 eV associated with OH is observable, together with a peak evident around 5.6 eV related to C=O bonding. Here SEHI demonstrates that it is possible to map functional groups on the surface of PGS-M using the components generated from nnmf.

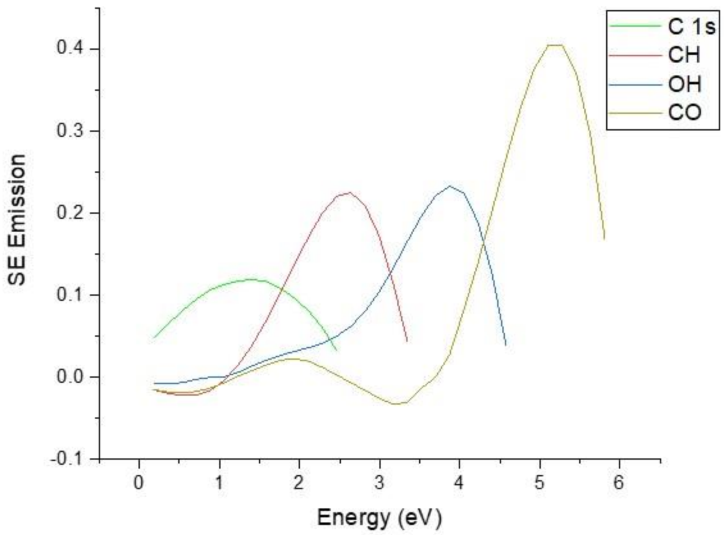

Figures S 7 - Non negative matrix factorisation (nnmf) multivariate analysis of AC, Ar 4 and Ar 10 treated PGS-M. Isolating four components.

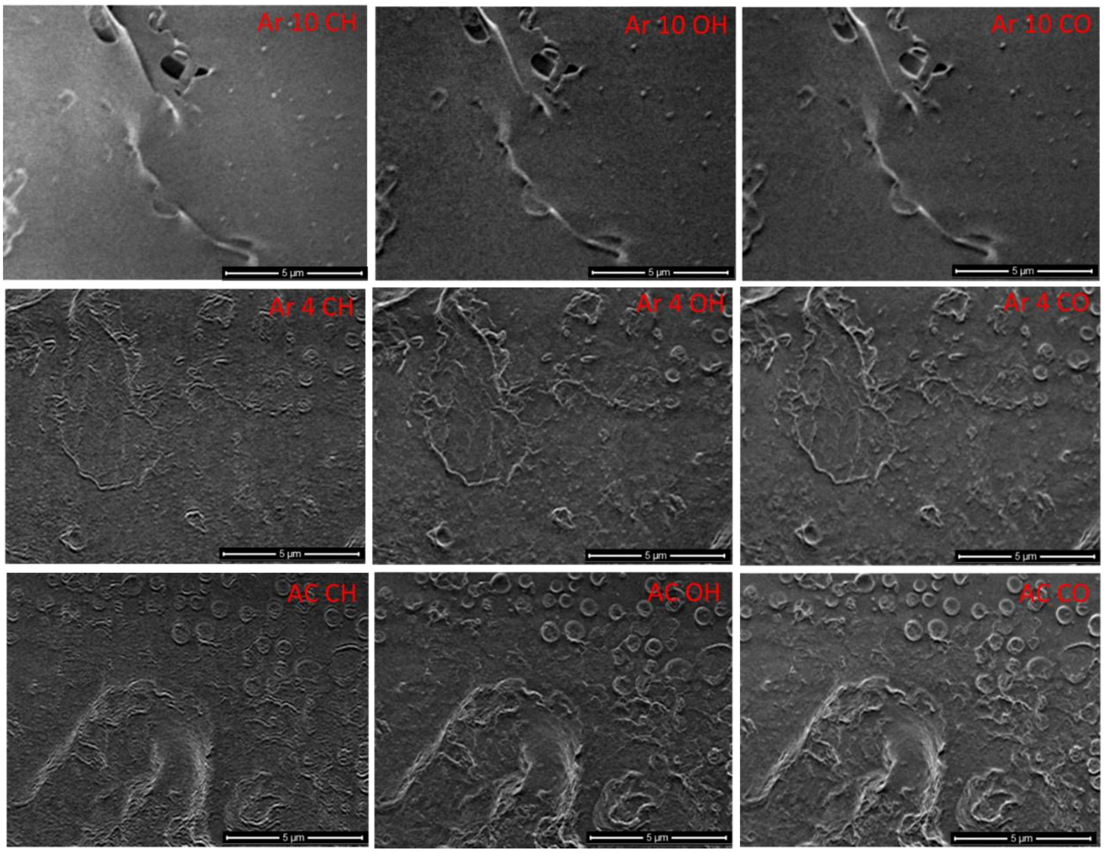

Figure S 8 - Resulting SEHI images of AC, Ar 4 and Ar 10 treated PGS-M. Isolating functional group components from non negative matrix factorisation (nnmf) component analysis.

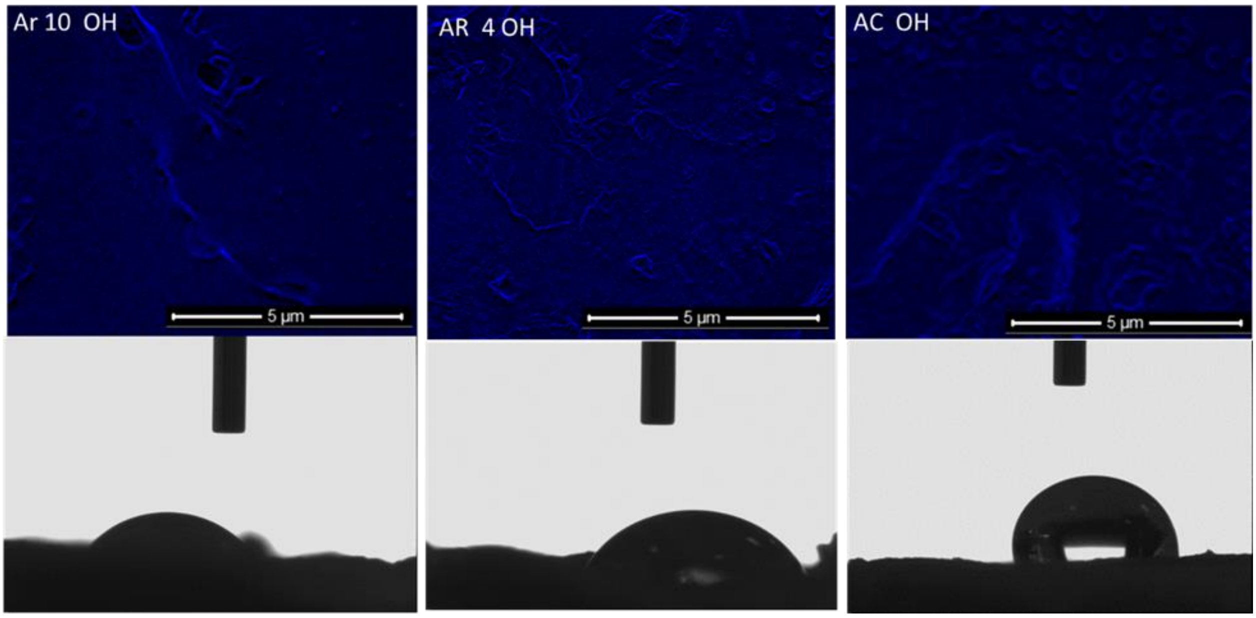

Figure S 9 - Resulting SEHI images of AC, Ar 4 and Ar 10 treated PGS-M. Isolating one component (OH) from non-negative matrix factorisation (nnmf) component analysis.

Analysis of Autoclaved PGS-M vs Non Sterile (Non treated) PGS-M

This publication focuses on PGS-M as a biomaterial source, therefore, there are no circumstances that can be conceived where it could possibly be implanted without first being subject to sterilisation. However, SE emission data was collected on non-treated non-sterile PGS-M as a comparison study with autoclaved PGS-M and is presented in Figure S10. Previous group publications (1) have discussed the development of PGS-M and the practicalities of the inclusion of methacrylate to render the polymer photo-curable. Producing this photocurable form of PGS gives the material a host of production benefits including the speed and simplicity of the singles step photocuring process and the potential ability to attach bioactive molecules on the remnant acrylate groups without the fear of denaturing as a result of heat polymerisation, However, as a non-heat sterilisation method was yet to be evaluated this previous work included autoclaving as a terminal sterilant.

As it is known PGS/PGS-M can be cross-linked with high temperatures (120°c for 48 hours) (12). The process of autoclaving is performed with the material being hydrated. The consequence of hydrating PGS-M was that the polymer would not crosslink in response to heat, as high pressures in heat polymerisation are required to remove water from the material. If the polymer is still hydrated this process cannot be completed. Therefore, the only crosslinking within the material would be in response to the UV photocuring step. Subsequent to the established process step of including dH2O to stop the mechanism of autoclaving causing cross-linking, SE spectra was collected before and after autoclaving. It was established that this process resulted in autoclaving inducing no extra crosslinking. Instead a slight decrease of CH vibrations (2.9 – 4.3 eV) previously linked to crosslinking density of the material (8) was observed. This was also coupled with a very slight overall emission decrease in molecular order (1.4 – 2.3 eV) of the polymer.

This decrease in cross-linking and molecular order associated emission was expected in response to hydrolysis degradation, both through the hydrolysis of anhydride, stopping further cross-linking, and the hydrolysis of the PGS-M backbone ethers (Figure S11). It is the design intent of PGS/PGS-M to degrade by ether hydrolysis within the body over time. This is common with thermally cured PGS which showed degradation in PBS, reducing in mass by 10% in 31 days (13). However, this process was advanced when high autoclave temperatures were used. Hydrolysing cross-linked PGS-M reduces CH vibrations as well as produce amorphous regions within the polymer surface. SE emission associated with OH/CO is increased as the formation of OH/CO containing functional side chains are formed through the ether hydrolysis reaction (Figure S11).

Figure 2 in the main manuscript displays how functional group distribution influences local plasma etching behaviour thus leading to topography and surface chemistry changes. As mentioned in the manuscript this isn't a novel finding for organic compounds as it is known plasma treatment of Polytetrafluoroethylene (PTFE) strips the fluorine molecules from the

carbon backbone of the polymer. Topographically, plasma treatment changes the surface morphology of PTFE with different morphologies resulting from different plasma gases used (14). The inset in S10 is a further indication of this process for PGS-M as it is clear non-treated PGS-M samples show micron-scale structures which appear to diminish after Ar plasma treatment (Figure 2). This is an expected effect of OH/CO containing sidechains from PGS-M being stripped away by Ar plasma changing the surface morphology and chemistry.

To allow a comparison to be drawn using SEHI images analysis, nnmf component analysis was performed on both AC PGS-M and non-treated PGS-M. The SE spectra components of both materials given from nnmf are shown in figure S12. Here we see components highlighted with peaks around 3.5 eV, 4.5 eV, and 5.5 eV. These peak positions fit into the ranges previously highlighted as CH, OH and CO functional groups emissions. Figure S13 allows the comparison of SEHI images produced from nnmf. SEHI images show that CH emission (given in red) has a slightly greater overall intensity within AC PGS-M than Non treated PGS-M. Whereas for CO and OH emissions, non-treated PGS-M has a greater emission than that of AC PGS-M.. These SEHI images corroborate the SE spectrums shown in S10. Figure S13 also includes an overlay of OH and CH SEHI maps for AC PGS-M. This overlay shows emission intensities from OH and CH differing across the materials surface. Future work should look to introduce colour mapping for SEHI images to allow multiple overlays without losing the high image resolution

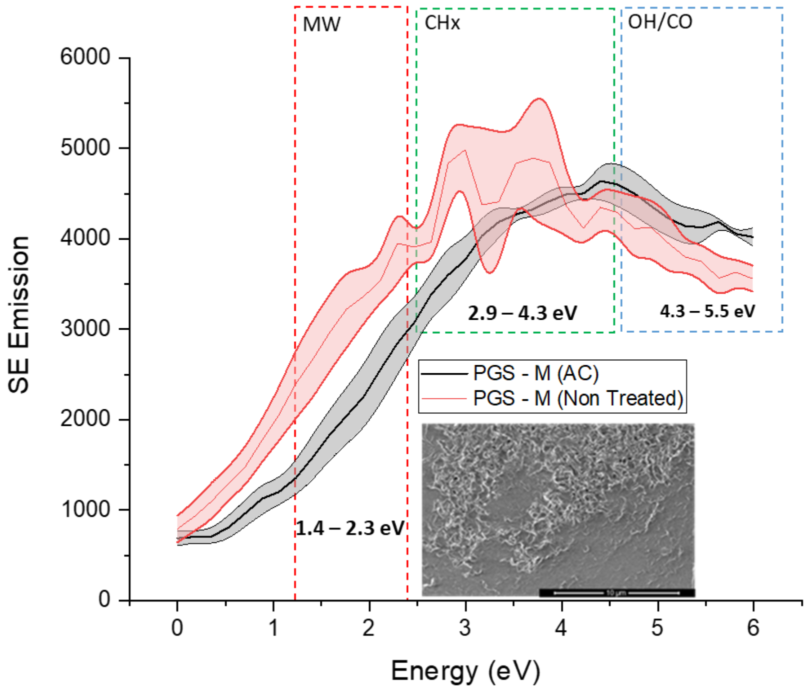

Figure S10 - Secondary electron spectra for PGS – M (non-sterile) and PGS-M after autoclave (AC) sterilisation. Inset shows an SEM image (HFW 15 um) of non-sterile PGS-M.

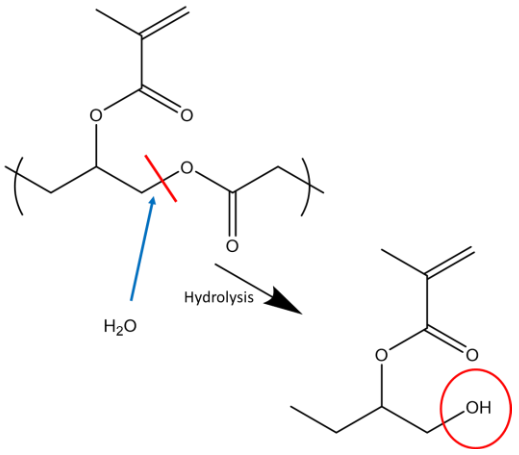

Figure S11 – Hydrolysis reaction of PGS-M.

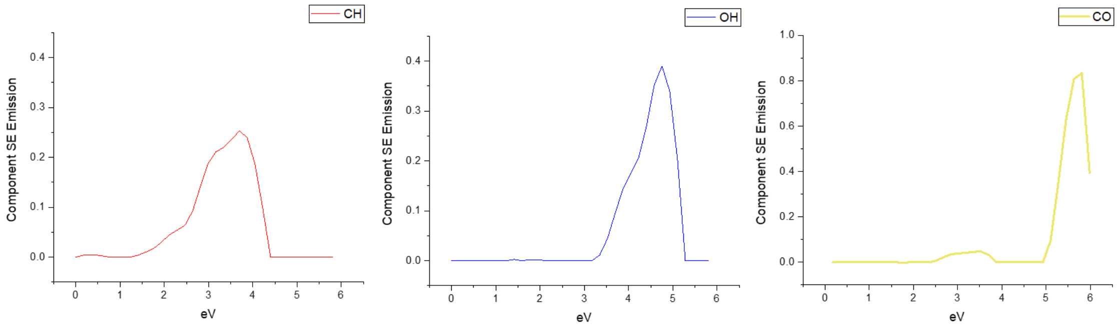

Figure S12 - Non negative matrix factorisation (nnmf) multivariate analysis of non-treated and AC PGS-M. Isolating three components.

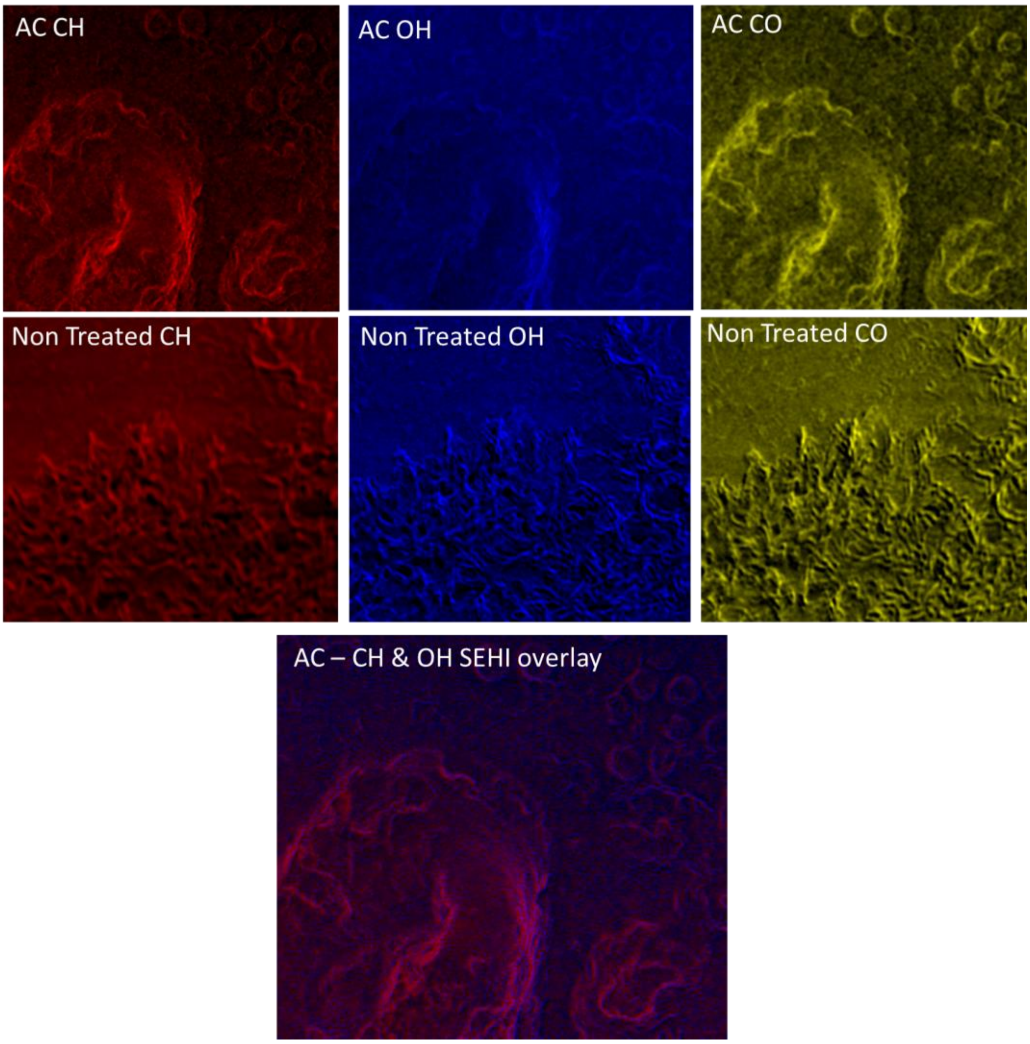

Figure S13 - Resulting SEHI images of non-treated and AC PGS-M. Isolating three components from non negative matrix factorisation (nnmf) component analysis. SEM images showing topography emission (>6 eV) are also included. HFW for all images shown is 4 um. SEHI emission intensities for AC images differ slightly to that in the main manuscript. This is as a result of nnmf analysing just the two conditions (non treated and AC) than that of all treatment groups.

<u>Using Secondary Election Spectroscopy to assess structural changes through topographical changes</u>

Figure 1B shows SEHI images of PGS-M after Ar 10, Ar 4 and AC treatments. These images indicate that AC PGS-M surface has an irregular topography compared to that of Ar 10 and Ar 4. In the main text of this publication we focus on emissions below 6 eV which are appropriate to functional group emissions and not surface morphology related SE emissions (9)(16) . However, it is known that analysing higher level SE energy emission can provide information regarding the topography of the material (15). Figure S14 corroborates SEHI images in Figure 1B by indicating a stronger SE emission in higher energies regimes of AC PGS-M compared to that of Argon plasma treatment samples. Future work to better understand this emission range would be beneficial but it is apparent that surface roughness can exhibit higher SE emission yields in higher energies. Figure S13 shows SEHI functional group component images accompanied with SEM images showing topography emissions greater than 6 eV. The previous section has discussed how functional group distribution influences local plasma

etching behaviour and as a consequence leads to topography changes. In figure S13 we see that topographical images show a depth perception and greater surface edge resolution compared to those of SEHI images. A caveat on this form of analysis is that different materials are expected to show chemical and topographical information at different energies as this information is dependent on the emission angle of the material (16).

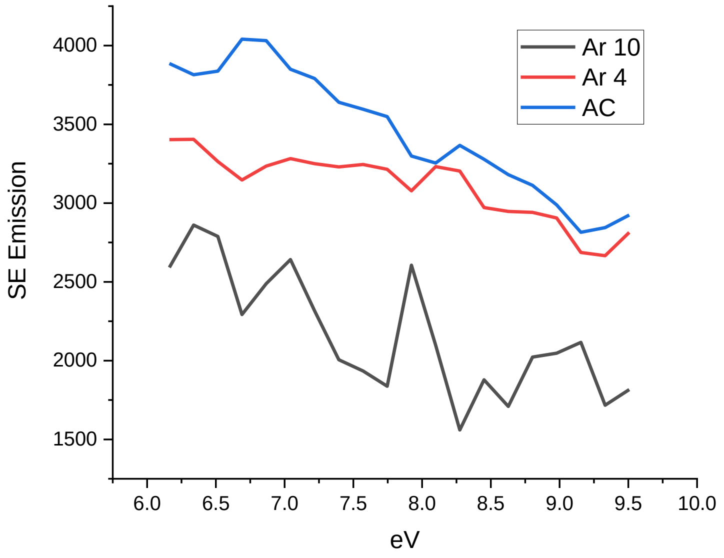

Figure S14 – Higher energies secondary electron spectra for PGS – M after undergoing AC, Ar 4 and Ar 10 plasma treatment.

References

1. S. Pashneh-Tala, R. Owen, H. Bahmaee, S. Rekštyte, M. Malinauskas, F. Claeyssens. *Front. Phys.* **2018.** *6*. 41.
2. J. Yip, K. Chan, K.M. Sin, K.S. Lau, *Mater. Res. Innov.* **2002**. 6, 44. 4.
3. P.S. Kazemian, A.M. Mentink, C. Rodenburg. CJ. Humphreys. Quantitative secondary electron energy filtering in a scanning electron microscope and its applications. Ultramicroscopy. 2007. 107, 140–150.

4. Q. Wan, K.J. Abrams, R.C. Masters, A.C.S. Talari, I.U. Rehman. F. Claeyssens, C. Holland, C. Rodenburg, A*dv. Mater*. **2017**. *29*, 1703510.
5. J. Schindelin, I. Arganda-Carreras, E. Frise, V. Kaynig, M. Longair, T. Pietzsch, S. Preibisch, C. Rueden, S. Saalfeld, B. Schmid, J.Y. Tinevez, D.J. White, V. Hartenstein, K. Eliceiri, P. Tomancak, A. Cardona, *Nat. Methods*. **2012**. *9*, 676.
6. N.A. Stehling, R. Masters, Y. Zhou, R. O'Connell, C. Holland, H. Zhang, C. Rodenburg, *MRS Commun*. **2018**. *8*, 226.
7. R.C. Masters, N. Stehling, K. Abrams, V. Kumar, A. Schäfer, D. Lidzey, C. Rodenburg, Adv Sci. 2019. 6, 5.
8. N. Farr, S. Pashneh-Tala, N. Stehling, F. Claeyssens, N. Green, C. Rodenburg. Macromolecular Rapid Communications. 2019. 41. 3
9. K.J. Abrams, M. Dapor, N. Stehling, M. Azzolini, S.J. Kyle, J.S. Schäfer, A. Quade, F. Mika, S. Kratky, Z. Pokorna, I. Konvalina, D. Mehta, K. Black, and C. Rodenburg. *Adv Sci*. **2019**. *6*, 19.
10. M. Abdouss, N. Sharifi-Sanjani, P. Bataille, *Journal of Applied Polymer Science*. **1999**. 74. 3417
11. K. Yuniarto, YA. Purwanto, S. Purwanto, BA. Welt, HK. Purwadaria, T. Candra Sunarti. *AIP Conference Proceedings*, **2016**. 1725, 020101
12. Y. Wang, GA. Ameer, BJ. Sheppard, R. Langer. A tough biodegradable elastomer. Nat Biotechnol. **2002**. 20:602–6.
13. M, Frydrych. B. Chen. Large three-dimensional poly(glycerol sebacate)-based scaffolds – a freeze-drying preparation approach. J Mater Chem B (2013) 1:6650–61. doi: 10.1039/C3TB20842G
14. R Kim-Sung. Journal of Applied Polymer Science. **2002**. 77: 1913–1920

15. V. Kumar, Vikas. W. Schmidt, G. Schileo, R. Masters, M Stringer, D. Sinclair, I. Reaney, D. Lidzey, C. Rodenburg, ACS Omega. **2017**. 2. 2126-2133.

16. Q. Wan, R. Masters, D. Lidsey, K. Abrams, M. Dapor, R. Plenderleith, S. Rimmser, F. Claeyssens, C. Rodenburg, Ultramicroscopy. **2016**. 171.
